# Plasma YKL-40 as a biomarker for bevacizumab efficacy in patients with newly diagnosed glioblastoma in the phase 3 randomized AVAglio trial

**DOI:** 10.18632/oncotarget.22886

**Published:** 2017-12-04

**Authors:** Mogens K. Boisen, Camilla B. Holst, Nicola Consalvo, Olivier L. Chinot, Julia S. Johansen

**Affiliations:** ^1^ Department of Oncology, Herlev University Hospital, Herlev and Gentofte Hospital, Copenhagen, Denmark; ^2^ Department of Medicine, Herlev University Hospital, Herlev, and Gentofte Hospital, Copenhagen, Denmark; ^3^ Biostatistics, F. Hoffmann-La Roche Ltd, Basel, Switzerland; ^4^ Department of Neuro-Oncology, Aix-Marseille University, Marseille, France; ^5^ Institute of Clinical Medicine, Faculty of Health and Medical Sciences, University of Copenhagen, Copenhagen, Denmark

**Keywords:** bevacizumab, YKL-40, newly-diagnosed, glioblastoma, AVAglio

## Abstract

YKL-40 is a glycoprotein with pro-angiogenic functions. We hypothesized that patients with newly diagnosed glioblastoma and low baseline plasma YKL-40 levels derive greater benefit from first-line bevacizumab. Plasma samples were collected from 563 patients in the randomized, phase 3 AVAglio trial who received bevacizumab or placebo plus radiotherapy/temozolomide. Raw plasma YKL-40 concentrations were converted to age-corrected percentiles of normal healthy YKL-40 levels and divided into quartiles (Q). The impact of baseline plasma YKL-40 level on survival was investigated using Cox regression analyses. Patients with low baseline plasma YKL-40 (≤Q1) had an improved progression-free survival hazard ratio (HR) for bevacizumab versus placebo (0.37, 95% confidence interval [CI]: 0.25–0.55) compared with high plasma YKL-40 (> Q1) (0.71, 95% CI: 0.57–0.87). Overall survival HRs were comparable between the subgroups (≤ Q1: 0.69, 95% CI: 0.44–1.09; (> Q1: 0.88, 95% CI: 0.68–1.13). A trend for improved progression-free survival HR with low versus high YKL-40 was observed in proneural glioblastoma (0.41, 95% CI: 0.13–1.28 vs 0.80, 95% CI: 0.45–1.40, respectively), but not for proliferative/mesenchymal subtypes. Elevated plasma YKL-40 (> 90th percentile of normal) was an independent negative prognostic factor. In conclusion, the predictive value of baseline plasma YKL-40 level as a biomarker for bevacizumab efficacy in glioblastoma may be limited to patients with proneural tumors. Independent validation studies are required to confirm these results.

## INTRODUCTION

*CHI3L1* (Chitinase-3-like protein 1) is one of the most overexpressed genes in glioblastomas, relative to normal brain and low-grade gliomas [1]. *CHI3L1* is a marker for the mesenchymal subtype of glioblastoma and is related to extracellular-signal-regulated kinases and protein kinase B phosphorylation [2]. YKL-40, which is encoded by the *CHI3L1* gene, is produced by tumor cells, inflammatory cells, and stem cells [3–5]. YKL-40 interacts with the receptors IL-13Rα2 [6], CRTH2 [7], RAGE [8], syndecan-1 [9], and PAR-2 [10], but its function is not completely known. YKL-40 induces vascular endothelial growth factor (VEGF) expression and both YKL-40 and VEGF are pro-angiogenic factors associated with tumor angiogenesis [11–17]. *In vitro*, YKL-40 up-regulates VEGF in the U87 human glioblastoma cell line, and long-term inhibition of VEGF induces expression of YKL-40 (potentially as a compensatory response, i.e. ‘angiogenic rebound’) [9, 11–17]. Surprisingly, shRNA knockdown of YKL-40 in tumor-derived mural-like cells (GSDCs) also increased VEGF expression and secretion [18]. These apparently contradictory results could be explained by tumor heterogeneity and cell diversity, different models with varying YKL-40 and VEGF expression, and a compensatory increase in VEGF as a result of long-term YKL-40 blockade in shRNA YKL-40 cells [18]. Irradiation of U87 cells increases YKL-40 expression [12, 19] and inhibition of YKL-40 enhances U87 cell death in response to irradiation [9]. *In vivo*, inhibition of YKL-40 in xenografted mice decreases angiogenesis, tumor formation, and metastasis in various tumor models [9, 12]. Of note, the expression of VEGF and YKL-40 are correlated in both xenografted mice and human glioblastoma samples [12]. Combination therapy with irradiation and anti-YKL-40 neutralizing antibody in xenografted glioblastoma tumor models resulted in decreased tumor growth, reduced tumor vascularization, and increased survival compared with monotherapy [14].

YKL-40 mRNA and protein expression increase with glioma grade and are higher in glioblastoma than in astrocytic- and oligodendroglial tumors [1, 2, 19–28]. In patients with glioblastoma, high YKL-40 expression in glioma cells is associated with poor response to radiotherapy (RT) and a shorter time to disease progression and death [14–17, 20, 21, 28]. YKL-40 is part of a 9-gene paraffin tissue-based expression panel that predicts survival in patients with glioblastoma [29]. Patients with epidermal growth factor receptor (EGFR) variant III-negative/YKL-40-negative tumors have a better prognosis than other tumor subtypes [25, 30]. It has also been shown that YKL-40 silencing in glioblastoma cells impairs proliferation, neurosphere formation, and their ability to induce endothelial cell migration [31].

In 55% to 75% of patients with glioblastoma, plasma YKL-40 is elevated compared with healthy subjects [32–34]. Iwamoto et al. showed that serum YKL-40 is lower in patients with glioblastoma and anaplastic gliomas without radiographic disease versus those with radiographic disease [33]. An increase in serum YKL-40 after surgery was also independently associated with short survival times in patients with glioblastoma or newly diagnosed anaplastic glioma [34].

Bevacizumab is a humanized monoclonal antibody directed against VEGF-A. Bevacizumab is often used as salvage therapy for patients with glioblastoma, either as monotherapy or in combination with different chemotherapy regimens [35]. A substantial proportion of patients appear to benefit from bevacizumab in this setting, and identifying this patient subgroup is an unmet need to date. In the AVAglio study, patients with newly diagnosed glioblastoma treated with RT and temozolomide (TMZ) in combination with bevacizumab had improved progression-free survival (PFS), but not overall survival (OS), compared with patients treated with RT/TMZ and placebo [36]. A similarly designed study confirmed the lack of OS benefit for bevacizumab in the first-line setting [37].

Objective response to bevacizumab has been correlated with improved OS in the recurrent setting [38], indicating that specific subgroups of patients may obtain a survival benefit from bevacizumab. Finding biomarkers that may identify patients eligible for bevacizumab treatment is therefore highly relevant. Based on the hypothesis that YKL-40 levels in blood and tumor correlate, and that low YKL-40 enhances the effect of bevacizumab (VEGF neutralization), we tested the hypothesis that patients in AVAglio with low YKL-40 plasma concentrations will derive a greater benefit from bevacizumab than patients with higher YKL-40 plasma concentrations, due to the proposed interaction between VEGF, YKL-40, and tumor angiogenesis [11–18].

## RESULTS

The biomarker-evaluable population comprised 279 patients treated with bevacizumab plus RT/TMZ and 284 patients treated with placebo plus RT/TMZ. Patient characteristics were comparable between the biomarker-evaluable and intent-to-treat (ITT) populations (Table 1). The first quartile (Q1), median, and third quartile (Q3) of raw plasma YKL-40 levels at baseline were 39 ng/mL, 65 ng/mL, and 109 ng/mL, respectively. The corresponding values for age-corrected percentile of normal plasma YKL-40 were 29th, 59th, and 89th percentiles. Raw- and age-corrected levels of plasma YKL-40 in the placebo compared with the bevacizumab cohort at baseline were: median 70 ng/mL *versus* 60 ng/mL (*p* = 0.02), and median percentile 70th *versus* 59th (*p* = 0.02), respectively.

**Table 1:** Comparison of clinical and biomarker-evaluable cohorts

	Total trial Cohort (*n* = 921), *n* (%)	Biomarker-evaluable Cohort (*n* = 563), *n* (%)	*p*^a^
Gender			0.91
Female	341 (37)	206 (37)	
Male	580 (63)	357 (63)	
Age			0.85
≤ 65 years	721 (78)	438 (78)	
> 65 years	200 (22)	125 (22)	
Race			0.72
Non-white	89 (10)	51 (9)	
White	832 (90)	512 (91)	
WHO PS			0.20
0	465 (50)	304 (54)	
1	455 (49)	258 (46)	
*MGMT* gene promoter status			0.39
Methylated	237 (26)	142 (25)	
Non-methylated	461 (50)	295 (52)	
Missing	223 (24)	126 (22)	
Type of surgery			0.22
Biopsy only	104 (11)	52 (9)	
Partial resection	433 (47)	284 (50)	
Complete resection	384 (42)	227 (40)	
Corticosteroid use at baseline			0.63
< 2 mg	522 (57)	327 (58)	
≥2 mg	395 (43)	232 (41)	
Missing	4 (<1)	4 (< 1)	
Smoking status			0.75
Current smoker	120 (13)	65 (12)	
Never smoker	470 (51)	292 (52)	
Past smoker	330 (36)	205 (36)	

^a^Fisher's exact test. Baseline characteristics with more than two levels were combined to compare the largest subgroup against all others.

MGMT = O-6-methylguanine-DNA methyltransferase.

### Baseline plasma YKL-40 and patient characteristics

Baseline plasma YKL-40 levels were higher in patients with poor World Health Organization (WHO) performance status (PS) and in patients who did not have a complete tumor resection (Table 2). Baseline plasma YKL-40 did not differ significantly between molecular subtypes.

**Table 2:** Baseline plasma YKL-40 and patient characteristics

	YKL-40, ng/mL		YKL-40, percentile^a^	
	Median	*p*^b^	Median	*p*^b^
Gender		0.40		0.28
Female	62		64	
Male	67		67	
Age		< 0.0001		0.07
≤ 65 years	57		64	
> 65 years	98		73	
Race		0.14		0.24
Non-white	90		78	
White	64		64	
WHO PS		< 0.0001		0.001
0	53		56	
1	78		73	
Type of surgery		0.03		0.0496
Biopsy only	65		64	
Partial resection^c^	69		70	
Complete resection	55		60	
Smoking status		0.31		0.93
Current smoker	57		60	
Never smoker	63		65	
Past smoker	70		67	
Molecular subtype^d^ (*n =* 305)		0.49		0.41
Proneural	67		61	
Mesenchymal	70		70	
Proliferative	61		63	

^a^Percentile of YKL-40 according to the established normal YKL-40 level in healthy individuals [39].

^b^*p* value from Mann-Whitney U test for two groups or Kruskal-Wallis test for > 2 groups comparing biomarker levels across the groups.

^c^Partial resection meant that some extent of tumor debulking was undertaken but macroscopically visible tumor was left behind. This should be contrasted with either diagnostic biopsy only or removal of all visible tumor (complete resection). Since patients with partial resections have larger tumor burdens, higher levels of YKL-40 are to be expected.

^d^Molecular subtype as defined in the study by Philips et al. 2006 [24].

### Elevated baseline plasma YKL-40 and prognosis

Fifty-six patients (10%) had elevated plasma YKL-40 (> 90th percentile of normal) at baseline, which was an independent predictor of shorter PFS and OS (Table 3). The estimated impact of elevated baseline YKL-40 (PFS hazard ratio [HR] 1.84 and OS HR 1.94) was comparable to the impact of poor WHO PS and non-methylated O-6-methylguanine-DNA methyltransferase gene promoter.

**Table 3:** Elevated baseline plasma YKL-40 and prognosis - multivariate analysis

Progression-free survival^a^			
	HR	95% CI	*p*
Treatment: BEV *versus* Pl^b^	0.60	0.48–0.76	< 0.0001
YKL-40 level: elevated *versus* normal^b^	1.84	1.20–2.80	0.0047
Age, per year increase	1.02	1.00–1.03	0.018
Race: white *versus* non-white	1.61	1.12–2.32	0.011
WHO performance status: 0 *versus* 1 to 2	0.69	0.55–0.87	0.0013
*MGMT*: methylated *versus* non-methylated	0.47	0.37–0.61	< 0.0001
Type of surgery: biopsy *versus* complete/partial resection	0.45	0.25–0.79	0.0058

Overall survival^a^			
	HR	95% CI	*p*
Treatment: BEV *versus* Plb	0.94	0.74–1.20	0.63
YKL-40 level: elevated *versus* normal^b^	1.94	1.23–3.06	0.0042
Age, per year increase	1.02	1.00–1.04	0.011
Race: white *versus* non-white	1.74	1.16–2.61	0.0076
WHO PS: 0 *versus* 1 to 2	0.66	0.52–0.84	0.0007
*MGMT*: methylated *versus* non-methylated	0.39	0.29–0.51	< 0.0001
Corticosteroid use at baseline: < 2 mg *versus* ≥ 2 mg	0.75	0.59–0.95	0.019

^a^Only significant variables are shown. The following variables were included in the multivariate model but were not significantly associated with either endpoint: gender, Mini-Mental State Examination score, delay between surgery and subsequent treatment, primary *versus* secondary glioblastoma, enzyme-inducing anti-epileptic drug use at baseline, and confirmation of glioblastoma histology (yes/no).

^b^Cut-off used for the definition of elevated plasma YKL-40 was the 90th percentile of normal [39].

BEV = bevacizumab; MGMT = O-6-methylguanine-DNA methyltransferase; Plb = placebo.

### Baseline plasma YKL-40 and bevacizumab efficacy

**Figure 1:**
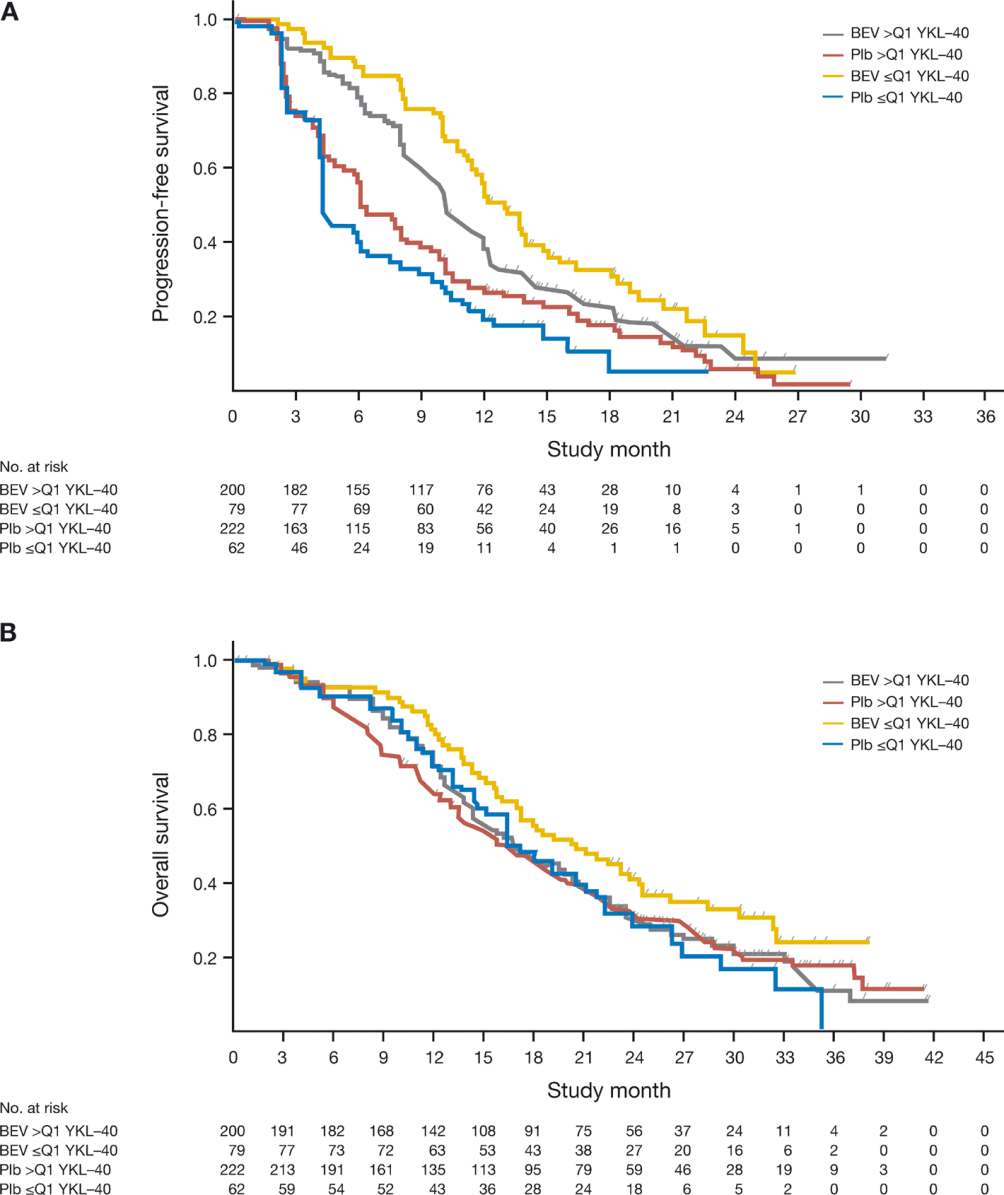
Kaplan-Meier survival curves for (**A**) progression-free survival and (**B**) overall survival, according to baseline plasma YKL-40 levels. BEV, bevacizumab; Plb, placebo; Q1, lowest quartile of plasma YKL-40 levels.

Bevacizumab was associated with longer PFS in patients with baseline plasma YKL-40 both below and above the lowest quartile (Q1). The PFS HR was improved in patients with baseline plasma YKL-40 below Q1 (Table 4), relative to the PFS benefit seen for patients with higher baseline plasma YKL-40. The PFS HR was 0.37 (95% confidence interval [CI]: 0.25–0.55) for patients with YKL-40 levels ≤ Q1, and 0.71 (95% CI: 0.57–0.87) for patients with YKL-40 levels > Q1. The OS HR was comparable between the subgroups, with overlapping CIs: OS HR was 0.69 (95% CI: 0.44–1.09) for patients with YKL-40 levels ≤ Q1 and 0.88 (95% CI: 0.68–1.13) for patients with YKL-40 levels > Q1. The PFS and OS Kaplan-Meier curves according to YKL-40 level ≤ Q1 *versus* > Q1 are shown in Figure 1. In a multivariate analysis with correction for other baseline characteristics, the interaction between YKL-40 level with Q1 as cut off and bevacizumab treatment was not significant (*p* = 0.24 for PFS and *p* = 0.32 for OS).

**Table 4:** Baseline plasma YKL-40 level and bevacizumab efficacy

Progression-free survival						
YKL-40 level^a^	Plb + RT/TMZ		BEV+ RT/TMZ		HR	95% CI
	Patients, *n*	Median, months	Patients, *n*	Median, months		
≤ Q1	62	4.2	79	12.9	0.37	0.25–0.55
> Q1 to ≤ Median	70	6.5	71	10.0	0.63	0.44–0.91
> Median to ≤ Q3	71	7.8	70	10.2	0.85	0.58–1.24
> Q3	81	5.7	59	10.0	0.65	0.45–0.94
> Q1	222	6.1	200	10.0	0.71	0.57–0.87

Overall survival						
YKL-40 level^a^	Plb + RT/TMZ		BEV+ RT/TMZ		HR	95% CI
	Patients, *n*	Median, months	Patients, *n*	Median, months		
≤ Q1	62	16.6	79	19.1	0.69	0.44–1.09
> Q1 to ≤ Median	70	17.5	71	17.6	0.83	0.53–1.30
> Median to ≤ Q3	71	18.1	70	16.8	1.05	0.67–1.65
> Q3	81	13.4	59	15.4	0.85	0.56–1.29
> Q1	222	15.8	200	16.8	0.88	0.68–1.13

^a^Percentile of YKL-40 according to the established normal YKL-40 level in healthy individuals [39].

BEV = bevacizumab; Plb = placebo; Q1 to Q3 = first to third quartile of age-corrected YKL-40 levels.

When all samples were combined, the relative change in plasma YKL-40 level from baseline to second cycle, or from baseline to the end of combination treatment, was not correlated with bevacizumab efficacy (Supplementary Figure 1, available online only).

For patients with the proneural subtype of glioblastoma, there was a trend for improved PFS HR with low (cut off Q1) *versus* high plasma YKL-40 levels (> Q1), although CIs overlapped: PFS HR 0.41 (95% CI: 0.13–1.28) *versus* 0.80 (95% CI: 0.45–1.40). A less pronounced trend was noted for OS HR in patients with the proneural subtype of glioblastoma: HR 0.50 (95% CI: 0.17–1.45) *versus* OS HR 0.69 (95% CI: 0.39–1.24), for ≤ Q1 *versus* > Q1, respectively. No correlation between YKL-40 levels and bevacizumab efficacy was noted in patients with proliferative and mesenchymal glioblastoma subtypes (Table 5). The sample size for these calculations was very small, as evidenced by the wide CIs.

**Table 5:** Baseline plasma YKL-40 and bevacizumab efficacy by molecular subtype^a^

Plasma YKL-40 level^b^	*n*	PFS HR	95% CI	OS HR	95% CI
**Proneural**					
≤ Q1	21	0.41	0.13–1.28	0.50	0.17–1.45
> Q1	63	0.80	0.45–1.40	0.69	0.39–1.24
**Proliferative**					
≤ Q1	20	0.56	0.21–1.51	1.05	0.40–2.72
> Q1	59	0.55	0.32–0.97	0.91	0.51–1.62
**Mesenchymal**					
≤ Q1	36	0.58	0.27–1.23	1.01	0.47–2.20
> Q1	106	0.64	0.4–0.97	1.10	0.71–1.70

^a^Molecular subtype as defined in the study by Philips et al. 2006 [24].

^b^Percentile of YKL-40 according to the established normal YKL-40 level in healthy individuals [39].

### Plasma YKL-40 during treatment and at progression

Median plasma YKL-40 levels did not change greatly during treatment or follow up (Supplementary Figure 2, available online only) and no apparent difference was noted in temporal YKL-40 levels between the placebo and bevacizumab cohorts. However, plasma YKL-40 was numerically lower at the time of progression compared with baseline, although the difference was not extensive (Supplementary Figure 3, available online only).

## DISCUSSION

To our knowledge, this is the first large prospective/retrospective biomarker study to investigate the prognostic and predictive value of plasma YKL-40 in patients with glioblastoma who received bevacizumab. Based on evidence suggesting a pro-angiogenic role of YKL-40 and interaction with VEGF [18], we hypothesized that low levels of plasma YKL-40 could be predictive of greater clinical benefit from anti-VEGF treatment. Based on raw plasma concentrations of YKL-40 fitted to age-corrected percentiles using the established normal YKL-40 level in healthy individuals [39], we report a greater numerical median PFS benefit with bevacizumab in patients with baseline plasma YKL-40 ≤ Q1 (below the 29th percentile of normal). We chose to divide the samples by quartiles of plasma YKL-40 levels instead of using a continuous scale because we did not expect the predictive value of plasma YKL-40 to be a continuous phenomenon. This was supported by the finding that patients with plasma YKL-40 above Q3 had a HR for bevacizumab *versus* placebo that was very similar to the HR for patients with plasma YKL-40 between Q1 and the median.

Using Q1 as a cut off in the subset of patients with available molecular subtyping data, we saw the same trend for an improved PFS HR in patients with baseline plasma YKL-40 ≤ Q1, but only in patients with the proneural subtype of glioblastoma. Although the results of this subgroup analysis are based on a small number of patients, this finding is encouraging, since these patients may be the ones who derive most benefit from front-line bevacizumab [40].

The differences observed were larger for PFS than for OS, which may have been confounded by second-line cross-over to bevacizumab in the placebo group (common in this study) [36]. However, cross-over did not influence the magnitude of bevacizumab benefit in patients with the proneural subtype of glioblastoma. In the present analysis, molecular subclasses were defined according to Phillips et al. [24], but other classifications exist [2, 41] and different subtypes have been found within the same tumor. This intra- and inter-tumor heterogeneity in glioblastoma complicates the analysis of potential correlations between molecular subtype and biomarkers like YKL-40 [41] and a more advanced subtyping could strengthen the use of YKL-40 as a biomarker for bevacizumab response. YKL-40 expression is a key feature of the mesenchymal glioma subtype and is also found in proneural tumors, where it has been located in tumor cells associated with blood vessels. In the description of the molecular sub-classification, Phillips et al. reported that eight of 26 matched tumors changed subtype at recurrence; seven of these to the mesenchymal subtype [24]. Depending on the significance of YKL-40 leakage from tumor tissue to systemic circulation and intratumoral heterogeneity, our results could imply that patients with proneural tumors and low plasma YKL-40 may have a better prognosis because neither systemic- nor tumor-related YKL-40 is affecting tumor growth or immune functions. To our knowledge, only Kazakova et al. [42] have described both tumor and serum YKL-40 in patients with glioblastoma (*n* = 14) and suggested that serum YKL-40 correlated positively with tissue YKL-40 expression, although it was not clear how this analysis was performed.

In patients with chemotherapy-refractory ovarian cancer treated with single-agent bevacizumab, low-to-normal plasma YKL-40 at baseline was associated with longer PFS and OS suggesting that plasma YKL-40 could be a predictive biomarker for bevacizumab in several tumor types [43].

Ninety percent of patients had normal plasma YKL-40 (< 90th percentile of normal) at baseline in the current study, which could be a reflection of the low tumor burden in these newly diagnosed patients. We validated the previously reported negative prognostic impact of having an elevated plasma YKL-40 at baseline. We did not find any predictive impact of change in plasma YKL-40 during treatment, but the power for this analysis was low.

Loss of blood-brain barrier integrity with ensuing changes in trans- and para-cellular transport is a characteristic associated with high-grade gliomas [44, 45] and is used to assess therapeutic response. In glioblastoma- and glioblastoma/oligodendroglioma-mouse models, weekly magnetic resonance imaging (MRI) scans showed increased blood-brain barrier disruption, which was incomplete only in the glioblastoma/oligodendroglioma model [45]. It is possible that this heterogeneous breakdown of the blood-brain barrier in glioblastoma releases variable amounts of YKL-40 into the circulation, making it difficult to interpret plasma YKL-40 as a biomarker, compared with cancers outside the central nervous system. A comparison of contrast-enhancement and perfusion on MRI and the corresponding plasma YKL-40 levels could further elucidate this subject. It would have been relevant to compare plasma YKL-40 levels with tumor tissue YKL-40 expression, measured using immunohistochemistry, but we did not have access to tissue samples in this study.

Among the strengths of this study was the large number of plasma samples collected (*n* = 563), although, some subgroup numbers were low. Furthermore, the samples were collected prospectively as part of a randomized, placebo-controlled trial and we tested a prespecified hypothesis that had a biologic rationale. Moreover, a trend for a predictive effect of plasma YKL-40 was seen only in the proneural molecular subtype, which has previously been suggested to derive most benefit from bevacizumab. Important limitations also exist; for example, the test for interaction between bevacizumab effect and baseline plasma YKL-40 with Q1 as a cut off was not significant in the multivariate analysis. This would be expected if the effect of YKL-40 was isolated to a smaller subset of patients, e.g. the proneural subset. It also suggests that baseline plasma YKL-40 alone is not a strong enough biomarker to accurately predict bevacizumab benefit. Furthermore, we did not provide an independent validation of our findings, and even though we prespecified a hypothesis about low plasma YKL-40 levels predicting a greater bevacizumab benefit, we did not lock a cut-off point before undertaking the study.

In conclusion, these results represent an encouraging first foray into the use of plasma YKL-40 as a biomarker in patients with glioblastoma treated with bevacizumab. Further studies are needed to investigate plasma YKL-40 in patients with cancer given an anti-angiogenic agent, to validate the positive predictive effect of low-to-normal plasma YKL-40 levels, and to determine the optimal cut-off point. It would also be of interest to investigate the double-inhibition of VEGF and YKL-40 for the treatment of cancer.

## MATERIALS AND METHODS

### Ethics statement

The protocol was approved by the applicable independent ethics committees and institutional review boards. All clinical investigations were conducted according to the principles expressed in the Declaration of Helsinki and according to national and international guidelines and were approved by the authors’ institutional review board. All patients provided written informed consent.

### Patients

AVAglio was a randomized, double-blind, placebo-controlled trial (NCT00943826; https://clinicaltrials.gov/ct2/show/NCT00943826) [36]. Full methods, including patient randomization, treatment, and follow-up, are published [36]. Patients with newly diagnosed glioblastoma received bevacizumab or placebo in combination with standard post-surgical RT/TMZ. Eligibility criteria included a WHO PS of ≤ 2, adequate healing of the operation site, and preserved organ function. Patients were excluded if they had a recent symptomatic intracranial hemorrhage, prior brain RT, prior glioblastoma treatment, recent intracranial abscess, or a serious non-healing wound. After surgical resection or biopsy, patients received RT consisting of 2-Gy fractions 5 days per week for 6 weeks, concurrent with daily oral TMZ (75 mg/m^2^), in combination with intravenous bevacizumab (10 mg/kg) or placebo every 2 weeks. Following RT/TMZ and a 4-week treatment break, patients received maintenance treatment with TMZ (150 mg/m^2^/day on days 1 to 5 during the first 4-week cycle and 200 mg/m^2^/day on days 1 to 5 during the following cycles) and intravenous bevacizumab (10 mg/kg) or placebo every 14 days for six 4-week cycles. Subsequently, patients without progression were treated with bevacizumab (15 mg/kg) or placebo as monotherapy every 3 weeks until progression or unacceptable toxicity [36].

The determination of progression was based on a combination of MRI, clinical assessment, and corticosteroid use, as previously described [36]. Co-primary endpoints were PFS, defined as time from randomization to progression or death from any cause, and OS, defined as time from randomization to death from any cause.

### Blood sampling and plasma YKL-40 analysis

The first plasma sample (baseline) was drawn within 29 to 48 days after surgery and before treatment with RT/TMZ and bevacizumab/placebo. Thereafter, plasma samples were drawn at regular intervals during treatment and at the time of disease progression.

Blood samples were centrifuged within 2 hours at 3,000 × *g* for 10 minutes at room temperature. Plasma was transferred into cryotubes and stored at −80°C. Plasma concentration of YKL-40 was determined in duplicate using a commercial enzyme-linked immunosorbent assay (Quidel, San Diego, California). The detection limit was 20 ng/mL, the intra-assay coefficient of variation (CV) was < 5%, and the inter-assay CV was < 6% [39, 46]. Plasma YKL-40 was determined at the Department of Medicine, Herlev University Hospital, Denmark and investigators were blinded to the treatment arms and clinical data.

The reference intervals for plasma YKL-40 were determined in 3,130 healthy subjects (1,837 women, 1,293 men) aged 21 to 84 years from the Danish general population (part of the Copenhagen City Heart Study) [39]. These subjects had no known disease at the time of blood sampling from 1991 to 1994, and remained healthy and alive during the 16-year follow-up period. The median plasma YKL-40 concentration in these 3,130 healthy subjects was 40 ng/mL.

### Molecular subtypes of glioblastoma

Three molecular subtypes of glioblastoma have been identified by gene expression analysis: proneural, proliferative, and mesenchymal [24]. Using tumor samples from the AVAglio trial, Sandmann et al. found that the molecular subtype of glioblastoma could be predictive for bevacizumab efficacy [40]. Therefore, data regarding molecular subtypes were included in our analyses.

### Statistical analysis

Raw plasma concentrations of YKL-40 were transformed to age-corrected percentiles using the established normal YKL-40 level in healthy individuals [39]. All YKL-40 levels referred to are percentiles of normal level unless explicitly called ‘raw’. Comparisons were made between quartiles of YKL-40 levels (> Q1 *vs* ≤ Q1); and between normal (≤ 90th percentile) and elevated (> 90th percentile) YKL-40 levels. The selection of Q1 as a cut off was supported by the plot shown Supplementary Figure 4 (available online only).

Fisher's exact test was used to compare baseline characteristics between the biomarker evaluable population and the ITT population. The Mann-Whitney *U* test (for two groups) or Kruskal-Wallis test (for > 2 groups) was used to compare YKL-40 levels by baseline patient characteristics. Kaplan-Meier methodology was applied for time-to-event endpoints. The associations between plasma YKL-40 level and PFS and OS were tested using univariate and multivariate Cox regression. All results were reported in accordance with REMARK guidelines [47].

## SUPPLEMENTARY MATERIALS FIGURES


