# Supplementary material for: Plasma YKL-40 as a biomarker for bevacizumab efficacy in patients with newly diagnosed glioblastoma in the phase 3 randomized AVAglio trial
Source: Oncotarget. 2017 Dec 4;9(6):6752–62. doi: 10.18632/oncotarget.22886 (PMC5805511; doi:10.18632/oncotarget.22886)
Supplement: Supplementary file 1 [file oncotarget-09-6752-s001.pdf]

Plasma YKL-40 as a biomarker for bevacizumab efficacy in patients with newly diagnosed glioblastoma in the phase 3 randomized AVAglio trial

SUPPLEMENTARY MATERIALS

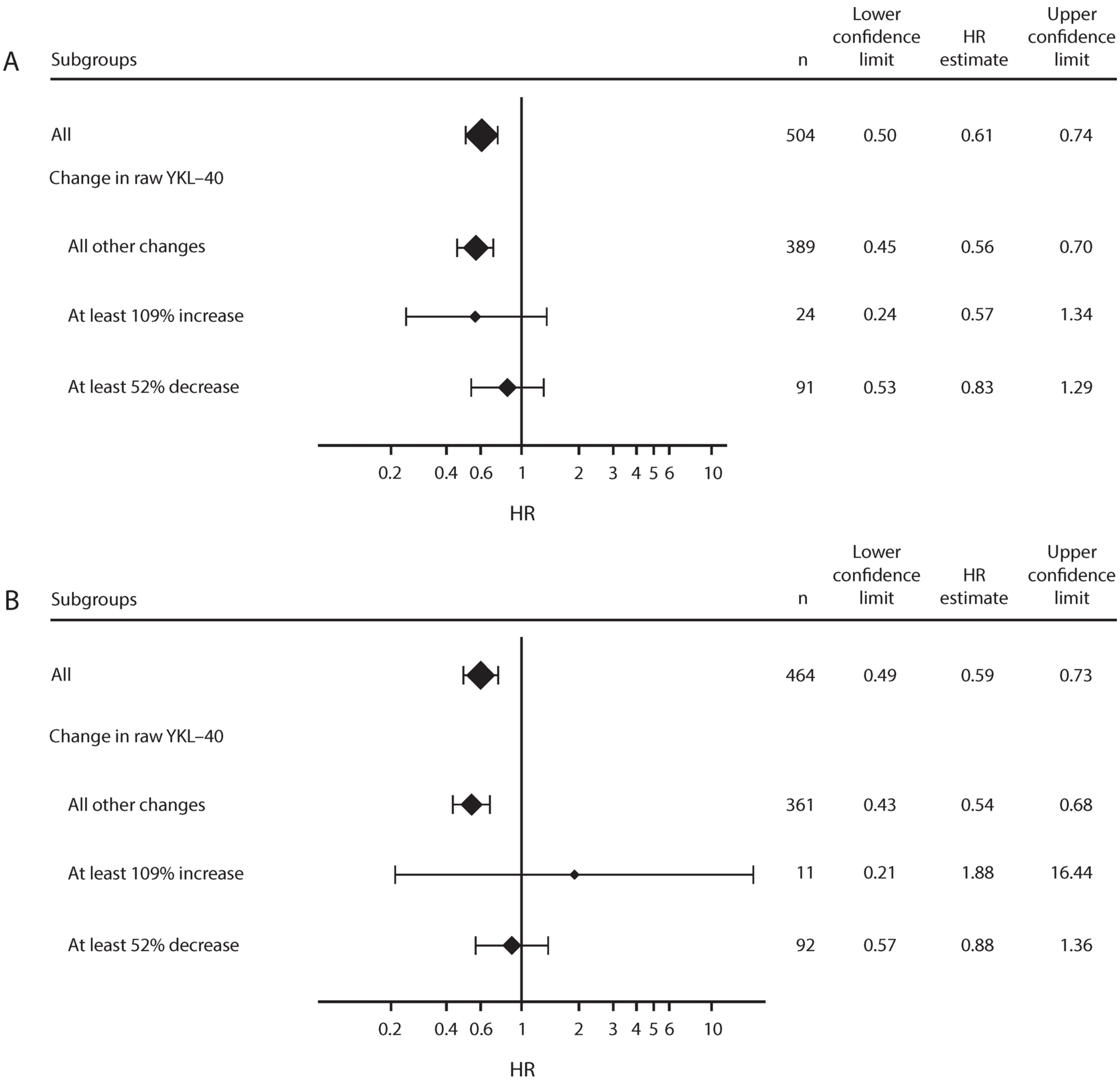

Supplementary Figure 1: Forest plot of progression-free survival according to relative change in raw YKL-40 level (**A**) from baseline to second cycle or (**B**) from baseline to the end of combination treatment. Abbreviation: HR, hazard ratio. The cut offs for percentage of change are based on prior studies of plasma YKL-40 measurement variability and correspond to the points where changes can be considered true changes [1].

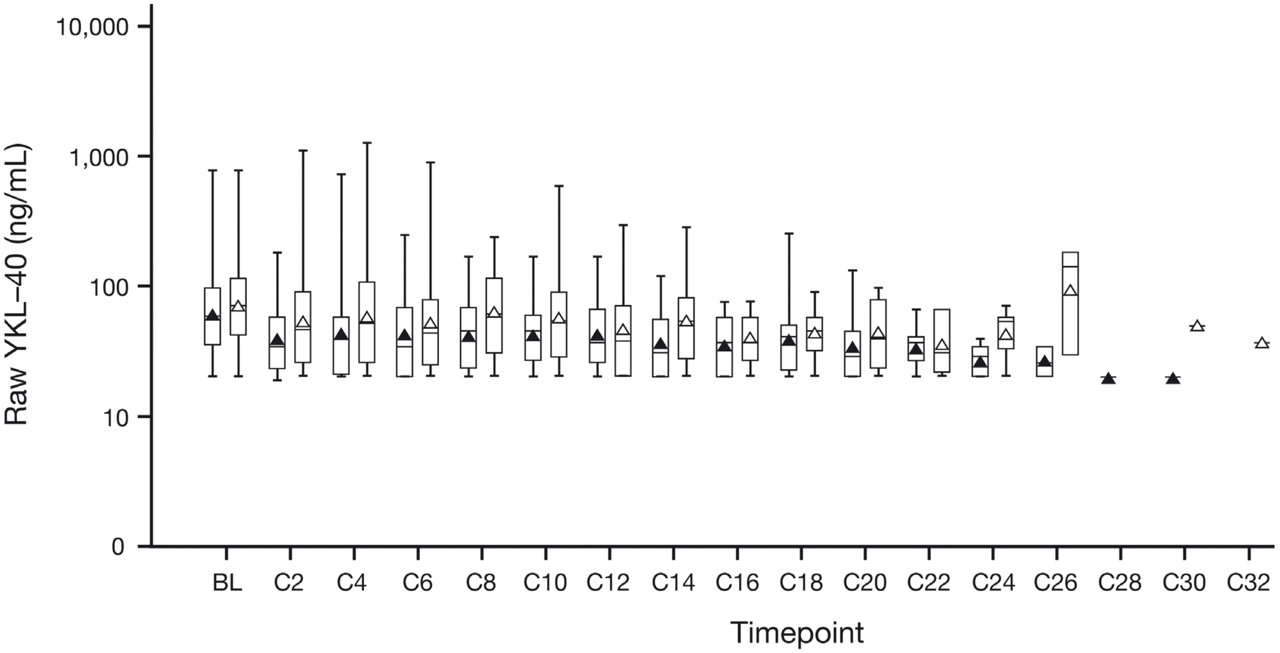

No. at risk

	BL	C2	C4	C6	C8	C10	C12	C14	C16	C18	C20	C22	C24	C26	C28	C30	C32
△ Plb + RT/TMZ	284	77	61	55	30	33	36	20	15	13	9	6	5	3		1	1
▲ BEV + RT/TMZ	279	133	102	96	47	37	36	24	23	16	11	7	6	2	1	1	

Supplementary Figure 2: Raw Plasma YKL-40 (ng/mL) at different time points during treatment and follow-up.
Abbreviations: BEV, bevacizumab; BL, baseline; C, cycle (monotherapy); Plb, placebo; RT/TMZ, radiotherapy and temozolomide.

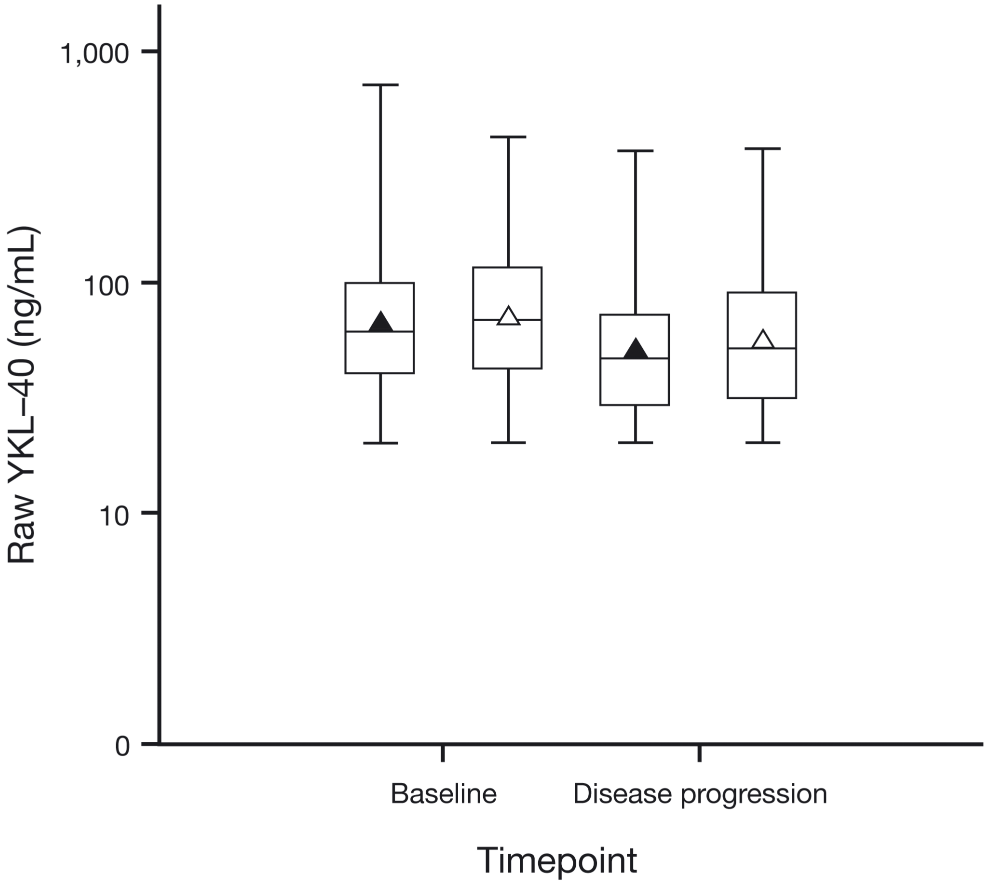

No. at risk

	Baseline	Disease progression
△ Plb + RT/TMZ	137	137
▲ BEV + RT/TMZ	89	89

Supplementary Figure 3: Raw Plasma YKL-40 (ng/mL) at baseline and at time of progression. Abbreviations: BEV, bevacizumab; Plb, placebo; RT/TMZ, radiotherapy and temozolomide.

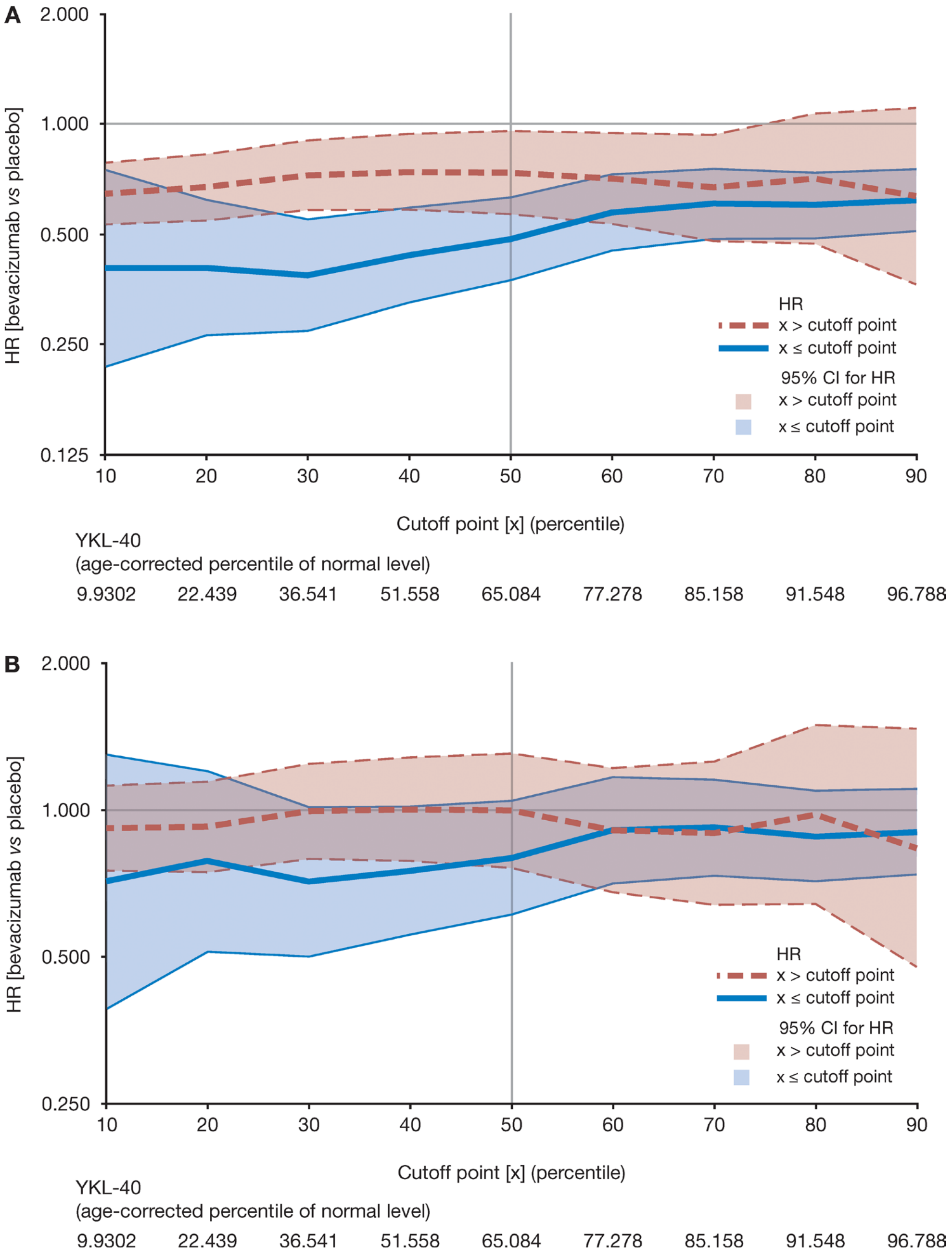

Supplementary Figure 4: Effect of bevacizumab on progression-free-survival (**A**) and overall survival (**B**) as a function of baseline plasma YKL-40 cut-off. Hazard ratios (HR) with 95% confidence intervals for the comparison between bevacizumab and placebo are shown for cohorts of patients with plasma YKL-40 above or below a given cut-off value of YKL-40. Population percentiles are shown on the x-axis and the corresponding age-corrected YKL-40 percentiles of normal value are shown below.

REFERENCES

1. Johansen JS, Lottenburger T, Nielsen HJ, Jensen JE, Svendsen MN, Kollerup G, Christensen IJ. Diurnal, weekly, and long-time variation in serum concentrations of YKL-40 in healthy subjects. Cancer Epidemiol Biomarkers Prev. 2008;17:2603–2608.
